# Unpublished systematic reviews and financial support: a meta-epidemiological study

**DOI:** 10.1186/s13104-017-3043-5

**Published:** 2017-12-06

**Authors:** Hiraku Tsujimoto, Yasushi Tsujimoto, Yuki Kataoka

**Affiliations:** 1 Hospital Care Research Unit, Hyogo Prefectural Amagasaki General Medical Center, 2-17-77 Higashi-Naniwa-Cho, Amagasaki, Hyogo 660-8550 Japan; 2 Department of Healthcare Epidemiology, Graduate School of Medicine and Public Health, Kyoto University, Yoshida Konoe-cho, Sakyo-ku, Kyoto, 606-8501 Japan; 3 Department of Nephrology and Dialysis, Kyoritsu Hospital, 16-5 Chuo-cho, Kawanishi, Hyogo 666-0016 Japan

**Keywords:** Publication bias, Meta-epidemiology, Systematic review, Registry, PROSPERO, PRISMA, Unpublished results, Dissemination bias

## Abstract

**Objective:**

PROSPERO, an international prospective register of systematic reviews, was launched in February 2011 to reduce publication bias of systematic reviews (SRs). A questionnaire survey of SR researchers conducted in 2005 indicated the existence of unpublished SRs and the potential influence of lack of funding as a reason for non-publication. Here, we investigated the publication status of registered SRs in the 1st year that PROSPERO launched and assessed the association between publication and the existence of funding or conflicts of interest (COIs).

**Results:**

We identified 326 SRs registered in PROSPERO from February 2011 through February 2012. Among them, 85 SRs (26%) remained unpublished at least 65 months after registration. We found 241 published reports, including four conference abstracts and one poster presentation. Median time to publication from protocol registration was 16.3 months. Funding for SRs was associated with publication [odds ratio (OR) = 2.10; 95% confidence interval (CI) = 1.26 to 3.50]. We found no significant association of author-reported COIs with publication (OR = 2.35; 95% CI = 0.67 to 8.20). Twenty SRs were not published despite the authors reporting completion of the reviews in PROSPERO.

**Electronic supplementary material:**

The online version of this article (10.1186/s13104-017-3043-5) contains supplementary material, which is available to authorized users.

## Introduction

Publication bias occurs when the publication of study results is influenced by the strength of the research findings. It includes two fundamental concepts: study findings and non-publication [1]. Many studies have reported bias in the dissemination of research findings other than systemic reviews (SRs) [2–4]. PROSPERO, an international prospective register of SRs, was launched in February 2011 to reduce publication bias of SRs [5]. Before PROSPERO, there was no specific international registration system for SRs; therefore, the issue of unpublished SRs could not be assessed directly. A questionnaire survey of SR researchers conducted in 2005 indicated the existence of unpublished SRs and the potential influence of lack of funding as a reason for non-publication [6]. As of June 2017, PROSPERO contains over 23,000 entries. Here, we investigated the publication status of SR protocols registered in PROSPERO and assessed the relationship of financial support for these SRs with their publication.

## Main text

### Methods

We investigated the publication status of registered SRs as of June 2017. The time frame for SR sampling was limited to the 1st year that PROSPERO launched in order to allow a lead time for publication. Cochrane has reported that the median time from protocol to SR publication is approximately 30 months [7]. We searched Google (https://www.google.com) and Google Scholar (https://scholar.google.com) in June 2017 for published SRs whose publication status was not reflected in the PROSPERO records. We defined publication as either author-reported publication status in PROSPERO, or dissemination of the results in a publicly available forum or in any journals indexed in Google or Google Scholar. We manually screened the search results first by titles and the URLs of all search results provided by Google on the results page and by titles of all search results provided by Google Scholar. If the website seemed to contain publication report, we entered the website and looked for full publication report. SRs that were not reported as published in PROSPERO or whose status was unavailable by searching Google or Google Scholar were considered unpublished. The search terms used for each SR were the title of the protocol, the PROSPERO ID, and the authors’ names. For example, we first searched Google Scholar using the PROSPERO ID. If we could not find the published report, we searched Google and Google Scholar using the title of the protocol enclosed in quotation marks. If this was unsuccessful, we searched Google and Google Scholar using the names of all listed authors of the protocol without quotation marks and screened the first page of search results. The time (in months) from protocol registration to publication of SRs was defined as the number of days from registration to publication divided by 30. The date of publication was selected for each review according to the following hierarchy: (1) the acceptance date, if available; (2) the online publication date, if available; (3) the earlier of the journal publication date or the date of author-reported “published” status in PROSPERO; (4) the earlier of the journal publication date of a conference abstract or the documented date of public poster dissemination detected by searching Google or Google Scholar. If only the publication month was reported, the midpoint of that month (day 15) was set as the publication day.

The association between publication and the existence of funding or conflicts of interest (COIs) was investigated using multivariable logistic regression analysis. Adjusted variables were funding and COIs because a previous study indicated the potential influence of lack of funding as a reason for non-publication [6]. We also conducted a post hoc analysis for the SRs registered in PROSPERO from September 2011 to February 2012 (posterior half period). Statistical analysis was performed using StataSE version 13 (StataCorp, College Station, TX).

### Results

**Fig. 1:**
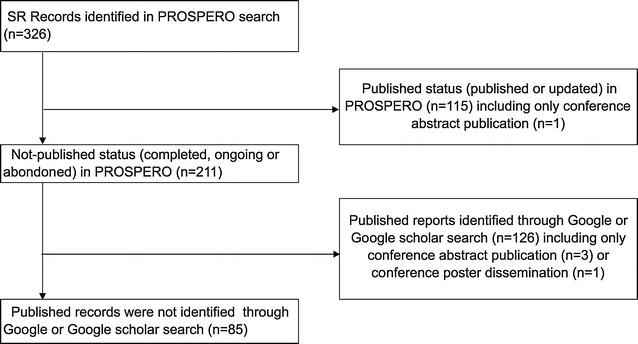
We first identified published SRs with published or updated status in PROSPERO records. One of them was only conference abstract publication. For 211 SRs with non-published status in PROSPREO, We investigated dissemination of the result in the publicly available space or published in any journals searched by Google or Google Scholar

**Fig. 2:**
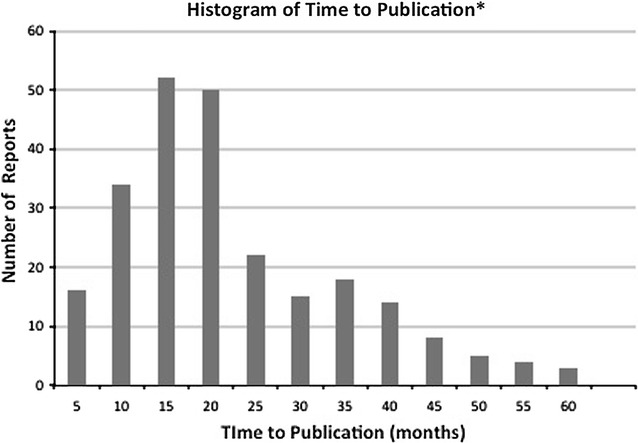
The minimum and maximum time to publication were 0.87 and 56 months. The median was 16.3 months. The average was 19.9 months. *The time (in months) from protocol registration to publication of SRs was defined as the number of days from registration to publication divided by 30. The date of publication was selected for each review according to the following hierarchy: (1) the acceptance date, if available; (2) the online publication date, if available; (3) the earlier of the journal publication date or the date of author-reported “published” status in PROSPERO; (4) the earlier of the journal publication date of a conference abstract or the documented date of public poster dissemination detected by searching Google or Google Scholar. If only the publication month was reported, the midpoint of that month (day 15) was set as the publication day

We identified 326 SRs registered in PROSPERO from February 2011 through February 2012. As Cochrane began registering protocols in October 2013, no Cochrane protocols were included [8]. The details of investigation of publication status are shown in Fig. 1. Among the identified SRs, 85 (26%) had not been published by June 2017, at least 65 months after protocol registration (the median time passed at the time of investigation was 68.8 months). We found 241 published reports, including 4 conference abstracts and 1 poster presentation. Of them, 126 SRs (52%) remained with unpublished status in PROSPERO. For SRs (N = 115) with published status, we could find published reports (100%). For 165 SRs (68%) we could find the exact accepted date for publication. For 25 SRs (10%), they reported the accepted date only by months; we used the mid-point of the month (day 15). For 51 (21%) SRs we could not find the accepted date, so we used the surrogate date according to the hierarchy described above. Median time to publication was 16.3 months (Fig. 2). Funding for SRs was associated with publication [odds ratio (OR) = 2.10; 95% confidence interval (CI) = 1.26 to 3.50; Table 1]. We found no significant association of author-reported COI with publication (OR = 2.35; 95% CI = 0.67 to 8.21; Table 1). The association was similar for the SRs registered in PROSPERO from September 2011 to February 2012 (Additional file 1: Table S1). Twenty SRs were not published despite the authors reporting completion of the reviews in PROSPERO.

**Table 1:** Results of multivariate logistic analysis for the publication of systematic reviews registered in PROSPERO

	Publication (n = 241)	Non-publication (n = 85)	Crude OR (95% CI)	Adjusted* OR (95% CI)
Funding^a^, n	170	45	2.13 (1.28 to 3.54)	2.10 (1.26 to 3.50)
COI^b^, n	20	3	2.47 (0.71 to 8.54)	2.35 (0.67 to 8.21)

*CI* confidence interval; *COI* conflict of interest; *OR* odds ratio; *PROSPERO* the international prospective register of systematic reviews

* Adjusted for funding and COI

^a^Funding sources/sponsors recorded in PROSPERO, which are defined as the details of the individuals, organizations, groups, or other legal entities who take responsibility for initiating, managing, sponsoring, and/or financing the review

^b^COI recorded in PROSPERO, which is defined as any condition that could lead to actual or perceived undue influence on judgments concerning the main topic investigated in the review

### Discussion

This is the first reported direct assessment of SRs remaining unpublished after protocol registration in PROSPERO and the relationship between publication and funding source. We found a considerable proportion (26%) of unpublished SRs even at more than 65 months after protocol registration. Tricco et al. reported that the publication of SRs may be affected by whether the results are informative [6]. We did not investigate the potential influence of the strength of research findings on publication (i.e., the effect of the clinical significance of SR results) because we could only access the published results retrospectively; however, these unpublished SRs might potentially impacted by the direction or strength of their findings.

Decullier et al. reported that funding was the determining factor for project initiation in clinical research, but once the project was initiated, funding had no significant influence on study completion or publication [9]. Conversely, we found that funding for SRs was related to their publication. One likely reason for these conflicting findings is the difference in funding sources between SRs and clinical trials: most SRs have non-profit funding [10], whereas clinical trials tend to have for-profit funding.

Non-profit funding for registered SRs may mitigate the issue of non-publication after protocol registration. Although we did not find a significant association between author-reported COI and publication, COIs reported by authors are actually a mixture of financial- and academic-related issues. Further research is needed regarding the association of financial conflicts of interest with SR publication.

## Limitations

The low proportion of SRs with registered protocols is a possible limitation of this study. Page et al. reported that only 16% of SRs published in 2014 have publicly available protocols [10]. Our sample included only SRs with protocols, which may not be representative of all SRs. However, the impact on assessment of non-publication of high-quality SRs was probably minimal, because the quality of reporting of published SRs in 2014 was still low despite the fact that reporting guidelines recommend protocol registration [10, 11].

Qualitative research by Tricco et al. revealed that the main reasons reported for non-publication were lack of time, overly broad SR scope, and few studies eligible for SRs as well as rejection [6]. Because data regarding these factors are unavailable in PROSPERO records, we could not adjust for them in our analysis, which is a limitation of this study.

In addition, while this survey covered only the 1st year after PROSPERO was launched, the proportion of protocol-registered SRs appearing in high-impact journals is increasing [12]. Future research should extend the search period to several years after launch in order to more fully investigate the characteristics of SR publications.

## Additional file

**Additional file 1: Table S1.** Results of multivariate logistic analysis for the publication of systematic reviews registered in PROSPERO from September 2011 to February 2012 (posterior half period).

## Abbreviations

PROSPERO: The international prospective register of systematic reviews
SR: systematic review
COIs: conflicts of interest
CI: confidence interval
OR: odds ratio
