# Supplementary material for: Unpublished systematic reviews and financial support: a meta-epidemiological study
Source: BMC Res Notes. 2017 Dec 6;10:703. doi: 10.1186/s13104-017-3043-5 (PMC5717810; doi:10.1186/s13104-017-3043-5)
Supplement: Supplementary file 1 — Additional file 1: Table S1. Results of multivariate logistic analysis for the publication of systematic reviews registered in PROSPERO from September 2011 to February 2012 (posterior half period). [file 13104_2017_3043_MOESM1_ESM.docx]

**Additional file**

Additional file Table S1: Results of multivariate logistic analysis for the publication of systematic reviews registered in PROSPERO from September 2011 to February 2012 (posterior half period).

|  | Publication  (n=137) | Non-publication  (n=55) | Crude OR  (95% CI) | Adjusted^*^ OR  (95% CI) |
| --- | --- | --- | --- | --- |
| Funding^†^, n | 97 | 29 | 2.17  (1.14 to 4.14) | 2.07  (1.08 to 3.95) |
| COI^‡^, n | 12 | 1 | 5.18  (0.66 to 40.87) | 4.51  (0.57 to 35.98) |

Abbreviations: CI = confidence interval, COI = conflict of interest, OR = odds ratio, PROSPERO = the international prospective register of systematic reviews.

*Adjusted for funding and COI.

†Funding sources/sponsors recorded in PROSPERO, which are defined as the details of the individuals, organizations, groups, or other legal entities who take responsibility for initiating, managing, sponsoring, and/or financing the review.

‡COI recorded in PROSPERO, which is defined as any condition that could lead to actual or perceived undue influence on judgments concerning the main topic investigated in the review.
